# Predicting Bystander Intention to Intervene: The Role of Gender-Specific System Justification and Rape Myth Acceptance for Men and Women

**DOI:** 10.3389/fpsyg.2020.00326

**Published:** 2020-03-13

**Authors:** Mara Martini, Norma De Piccoli

**Affiliations:** Department of Psychology, CIRSDe, University of Turin, Turin, Italy

**Keywords:** rape myth acceptance, gender-specific system justification, bystander intention to intervene, mediation model, gender differences

## Abstract

The relationship between rape myth acceptance, gender-specific system justification (GSJ), and bystander intention to intervene has often been studied on a one-dimensional basis, without separating the four dimensions of the acceptance of rape myths. The current study analyzes the relationship between the acceptance of rape myths, GSJ, and bystander intention to intervene, and explores whether the relationships operate differently for men and women. The sample was 3,966 university students: 2,962 from the University of Turin and 1,004 from the Politecnico of Turin; 71.2% women and 28.8% men; average age of 22.61 years. After descriptive analyses, independent sample *T*-test, and bivariate correlations, a model where the acceptance of four rape myths (“She asked for it”; “He didn’t mean to”; “It wasn’t really rape”; “She lied”) mediated the relationship between GSJ and bystander intention to intervene was tested on the whole sample and then separately on women and men. A bootstrapping procedure was applied. Our data show that for both men and women, GSJ was related to the four rape myths, whereas women and men differed on the relationship between acceptance of rape myths and bystander intention to intervene: only the dimension “She asked for it” was significant for both groups; the dimension “It wasn’t really rape” was significant only for the men. Focusing on the differences in women and men regarding acceptance of rape myths can be fruitful for a theoretical deepening of the field and may inform the development of more successful prevention programs.

## Introduction

Questions about sexual violence ask why there is a proclivity to underestimate the phenomenon, why it is perceived as a “private” issue, and, perhaps most importantly, why it is sometimes justified by either the perpetrator’s psychological fragility or assignment of the blame to the victim (VicHealth, 2014; Powell and Webster, 2018). Several studies (McMahon, 2010; Ståhl et al., 2010; Banyard, 2011; Diener, 2016) analyzed the reasons why these phenomena are reproduced and why they are difficult to counteract. In the analysis of attitudes of legitimation and underestimating of sexual violence, research has focused on rape myth acceptance (McMahon, 2010; Edwards et al., 2011; Joseph et al., 2013). From a psychosocial perspective, rape myths are defined as “attitudes and beliefs that are generally false but are widely and persistently held, and that serve to deny and justify male sexual aggression against women” (Lonsway and Fitzgerald, 1995, p. 134; Payne et al., 1999, p. 29). From early studies (Payne et al., 1999) through to today, various measurement scales have been developed. Several different kinds of instruments were required because, both theoretically and empirically, it was necessary to update the content and the language surrounding these beliefs (McMahon and Farmer, 2011). Rape myths are culturally based, and their forms of expression have evolved: scholars now use the term “subtle” rape myth to describe their existence, although the language is not so overtly “against the victim.” McMahon and Farmer (cit.) examined four forms of rape myths: “She asked for it”; “He didn’t mean to”; “It wasn’t really rape”; and “She lied,” Two assign blame to the victim: “She asked for it” and “She lied.” A woman “asked for it” with her provocative clothing or behavior, so if she had adequately covered her body, then no one would have bothered her. Among the different expressions that rape myth acceptance can have, Rollero and Tartaglia (2019) showed that particularly the myth “She asked for it” has “encouraged the attribution of blame to the woman” and thus “decreased the perception of the man’s responsibility” (p. 216). A “woman lies” when she intentionally fabricates the rape, for example, to take revenge for an affective relationship that did not have the outcome she had hoped for. The two other rape myths express an attitude to attenuate the cause and the consequences of rape: “He didn’t mean to” implies, for example, that male sexual instinct cannot always be kept under control, so if a man commits rape, he is not really at fault. “It wasn’t really rape” denies that sexual assault occurred, with the effect of either blaming the victim or excusing the perpetrator. Endorsement of rape myths serves to justify and minimize the harm of sexual abuse. Such minimization reduces the social stigma of sexual violence and renders measures against it less effective (Hinck and Thomas, 1999; Payne et al., 1999; Edwards et al., 2011). Moreover, rape myth acceptance is associated not only with a lower tendency to disclose sexual assault but also, inevitably, with a lower propensity to intervene against it (McMahon, 2010), partly because the assault is not recognized as illegitimate.

Recognizing (or not being able to recognize) sexual violence as illegitimate has its roots in psychosocial theories about dominant and subordinate groups. To ensure the maintenance of privileges acquired over time and to protect the *status quo*, “people create ideologies that legitimize the dominant group’s superiority, the subordinate group’s inferiority, and the reasons for systemic violence” (Jost and Banaji, 1994; Jost and Hunyady, 2002; Jost et al., 2003; Chapleau and Oswald, 2013, p. 19). This phenomenon involves gender relations as well, with males in the dominant position and females as subordinate group. Jost and Banaji (1994) developed the concept of gender-specific system justification (GSJ) to explain the persistence (and legitimation) of unfairness in gender relations and how the socio-cultural constructions of gender inequality are made to appear normal and just. According to the theory of system justification (Jost and Banaji, 1994; Jost and Kay, 2005), all members of a society identify to a certain extent with the current culture (the *status quo*). In the same way, social culture is a constituent component of one’s identity. Thus, even if this culture favors one group over another, both the dominant (males in the gender system) and the subordinate group (females) tend to defend it, both for its fair and less fair expressions (Jost and Banaji, 1994; Chapleau and Oswald, 2013). This happens also when a change could improve the conditions of the subordinate group. The theoretical analysis presented by Joseph et al. (2013) describes the link between system justification theory and the mechanisms underlying sexual assault. They state that “the motivation for system justification clearly plays a role in the general appraisal of sexual assault against women as an unwanted yet tolerated part of the social system. Therefore, efforts aimed at the prevention of sexual assault need to contend with this force” (p. 499). They also state that “if victims do choose to disclose their assault to sources of social support, they risk being accused of fabricating or somehow causing the experience as a result of the listener’s system justification efforts (Ståhl et al., 2010, p. 496).” Recognizing the seriousness of sexual violence, and persecuting the rapist after a disclosure, can in some way threaten the *status quo*: if the system of male dominance tolerates sexual assaults, prosecuting the perpetrator means affirming that the cultural system is unjust and therefore should be changed. Blaming the victim, on the contrary, preserves the *status quo* and listeners’ cultural identity (Chapleau and Oswald, 2013).

Rape myth acceptance and system justification gender orientation are socially developed, and they drive behaviors and social practices. Attitudes and practices are interdependently related: attitudes affect practices, “which in turn are associated with violence against women and hence are among the targets of interventions to prevent the problem” (Our Watch, 2015-2016; Powell and Webster, 2018, p. 43). The aim of sexual assault interventions is to change attitudes through education and training, modify gender stereotypes, raise awareness about equal opportunities and rights, and condemn all forms of sexual and sexist attitudes and behaviors.

Among the interventions to prevent sexual violence, a widely explored approach is bystander focused (Banyard, 2011; Ferrans et al., 2012; Banyard, 2015). The bystander is someone who witnesses a violent act; through his/her presence and intervention, the bystander can either prevent or stop the violent act (or at least offer a first response) or choose not to intervene. The bystander, to which Banyard (2011) refers in presenting this approach to preventing violence, (usually) does not have a specific role or training to counteract violence; the bystander may be a companion, a friend, a simple passerby, or a neighbor and may be present on the scene when the assault occurs and when the authorities are absent. One of the central aspects of the approach centered on the attitudes toward bystander intervention is the shift of responsibility to the community or to all community stakeholders.

The reason for bystander non-reaction has been explained since Darley and Latanè’s (1968) paper. Understanding the factors that can support or reduce bystander intention is crucial for encouraging individuals to intervene when needed (McMahon, 2010). There are numerous reasons why people intervene or not in a risky situation (Burn, 2009; Bennett et al., 2014; Pettit et al., 2017). Some factors that seem to foster the attitude to intervene in sexual assaults are related to the perception of the situation. Bystanders’ inclination not to intervene in cases of sexual violence may be related *inter alia* to the belief that the victim provoked the situation and is blameworthy or to the belief that the aggressor has no intention to harm the victim (Bennett et al., 2014; Johnson, 2015; Diener, 2016). The attribution of blame to victims of sexual assault or the underestimation of aggressors’ responsibility is related to acceptance of rape myths (McMahon and Farmer, 2011). Bystanders also may be affected by a social climate that legitimates gender discrimination. Such a climate represents GSJ (Joseph et al., 2013). In other words, two processes affecting the attitude to intervene are rape myth acceptance (McMahon, 2010; Johnson, 2015; Diener, 2016) and GSJ (Joseph et al., 2013). Both minimize the harm of sexual violence, as hypothesized by Joseph et al. (2013). This hypothesis, notwithstanding, has only been theoretically expressed and not yet empirically tested.

Moreover, as gender is a central point in this reflection, it is necessary to examine what the gender differences are in attitudes toward sexual assault and victims of sexual assault. Some scholars have analyzed these constructs and focused on gender differences. According to Grubb and Harrower (2008), women tend to assign blame to the victim less than men; this discrepancy, verified by many studies, indicates gender-related differences in rape myth acceptance (Krahé et al., 2007; Bannon et al., 2013; Bendixen and Kennair, 2017; Russell and Hand, 2017; Navarro and Tewksbury, 2017). In Western and Eastern societies, women accept rape myths less often than men, as several studies have highlighted (e.g., Kamdar et al., 2017, for India; Stephens et al., 2016, for Japan and India; Xue et al., 2019, for China). Since sexual assault is more commonly perpetrated by men, males and females show likely different grades of rape myth acceptance, higher for males. A man might justify the behavior of another member of his group, underestimating the offender’s responsibility. The woman, on the contrary, might identify herself with the victim, have a more “compassionate” attitude toward the victim, and be less likely to justify the act.

The study of Chapleau and Oswald (2014) is one of the few to consider, besides other psychosocial dimensions, the relationship between rape myth acceptance, GSJ, and gender differences. Their data show that men held (marginally) higher levels of GSJ and rape myth acceptance than women, and that for women, the level of GSJ was just as predictive of higher rape myth acceptance as for men. These findings suggest that for women, rape myths can be expressions of system justification. While women usually endorse rape myth acceptance less than men, they do not completely reject rape myths, because of their identification with a culture that more or less openly express the belief that men have a right to a dominant position and that “if a man sexually attacks a woman, it is because of her failure to comport herself around me” (Chapleau and Oswald, 2014, p. 213). Papp and Erchull (2017) demonstrated a positive relation between GSJ and rape myth acceptance in a sample of women; their study used the one-dimension scale of Payne et al. (1999), without distinguishing the different kinds of rape myths. To our knowledge, no previous studies have investigated jointly the relationships between gender, GSJ, and the four dimensions of rape myth acceptance.

### Current Research Aims and Hypotheses

The present study had three principal aims. The first was to analyze the relationships between GSJ, dimensions of rape myth acceptance, and bystander attitude toward intervention. Previous studies, indeed, have suggested that bystander intention to intervene is predicted by the degree of acceptance of false beliefs regarding sexual violence: if people think that women are responsible for being raped or think that some forms of sexual assault are not really serious, they are probably less committed to preventing sexual assault. Furthermore, rape myth acceptance can be understood as part of a cultural framework that justifies women’s subordinate position in a society. In other words, the attitude of acceptance of rape myths, which can, in turn, be predicted by the GSJ (e.g., Papp and Erchull, 2017), can influence bystander disposition to intervene (or not) in an act of sexual violence (McMahon, 2010; Johnson, 2015; Diener, 2016). According to Joseph et al. (2013), therefore, these different dimensions are all related to each other, and it can be assumed that rape myth acceptance mediates the relationship between GSJ and bystander intention to intervene.

The second aim was to explore whether the four dimensions of rape myth acceptance play different roles in these relationships. As they refer to different (false) representations of sexual assault, the different sub-dimensions of rape myth acceptance in these relations need to be considered distinctly. Taschler and West (2017) indeed suggested that “future research could examine specific types of rape myths in more detail rather than measure rape myth acceptance as a general variable” (p. 481).

Moreover, the third aim was to understand whether the relationships among the dimensions under study differed between women and men. In spite of the pervasive socio-cultural influence, and probably due to a tendency of women to identify with the victim, indeed, men and women differ in GSJ, rape myth acceptance, and their attitude toward intervention, as previous studies have shown in other contexts (Krahé et al., 2007; Bannon et al., 2013; Bendixen and Kennair, 2017; Emmers-Sommer, 2017; Russell and Hand, 2017; Navarro and Tewksbury, 2017).

Specifically, we wanted to verify the following hypotheses:

H1 – Women and men differ in how they perceive GSJ, the four dimensions of rape myth acceptance (“She asked for it”; “He didn’t mean to”; “It wasn’t really rape”; and “She lied”) and bystander intention to intervene. More precisely, we expected the following: (a) Women hold a higher level of bystander intention to intervene. (b) Men show a higher level both of GSJ and of the four dimensions of rape myth acceptance.
H2 – (a) The four dimensions of rape myth acceptance are positively related to each other. (b) GSJ is positively related with all four dimensions of rape myth acceptance. (c) GSJ is negatively related to bystander intention to intervene. (d) All four dimensions of rape myth acceptance are negatively related to bystander intention to intervene.
H3 – The influence of GSJ on bystander intention to intervene is fully mediated by the four dimensions of rape myth acceptance: specifically, GSJ has a positive impact on the four dimensions of rape myth acceptance, and all four dimensions of rape myth acceptance have a negative impact on bystander intention to intervene; no direct negative impact is present by GSJ on bystander intention to intervene.

Finally, we sought to answer the explorative question of how the hypothesized mediation model performed in the female and the male groups. Because of the inconsistencies among studies on victim blame (Gravelin et al., 2019), and because previous studies did not consider the four dimensions of rape myth acceptance, our aim was to further describe gender differences in the mediation model.

## Materials and Methods

### Participants

A total of 4,056 students responded to our online survey; 90 responses were discarded because they were incomplete. The breakdown of the remaining 3,966 questionnaires was as follows: 2,962 respondents from the University of Turin and 1,004 from the Politecnico of Turin; 71.2% were female, and 28.8% were male; 32.8% were in their freshman year; the average age was 22.61 ± 4.59 (SD) years; 94.8% were not married, and 98.5% had no children; and 56.8% lived with their parents, 26.4% with a roommate, 7.1% alone, 3.6% in a student dormitory, and 6.1% with a partner and/or child.

### Measures

The survey questionnaire included sociodemographic items (gender, age, year of university enrollment, marital status, having children, place of residence) and variables measured on the following scales:

– *System Justification* – *Gender Scale* (GSJ; Jost and Kay, 2005; our translation for this study). This tool consists of eight items on a Likert scale from 1 (“strongly disagree”) to 9 (“strongly agree”). The scale measures the extent to which respondents believe that the system of gender relations is equal and fair in their cultural and social context. For example: “In general, relations between men and women are fair.”
– An Italian adaptation (SRMA-IT) by Martini et al. (under review)^1^ of the *Updated Measure for Assessing Subtle Rape Myth* (McMahon and Farmer, 2011). This tool is composed of 20 items that assess the extent to which rape myths are shared by respondents. The Likert scale ranges from 1 (“strongly disagree”) to 5 (“strongly agree”) and measures factors that express the four myths: (1) “She asked for it” (six items, e.g., “When girls go to parties wearing provocative clothes, they are asking for trouble”); (2) “He didn’t mean to” (four items, e.g., “When guys rape, it is usually because of their strong desire for sex”); (3) “It wasn’t really rape” (five items, e.g., “A rape probably didn’t happen if the girl has no bruises or marks”); and (4) “She lied” (five items, e.g., “Rape accusations are often used as a way of getting back at guys”).
– The *bystander intention to intervene scale* (our adaptation for this study from Banyard et al., 2005). This tool, aimed at investigating bystander intention to intervene against sexual violence, is made up of 14 items describing actions against gender violence. In our version, respondents are asked to express on a Likert scale from 0 (“not confident at all”) to 10 (“completely confident”) to what extent they would put into practice the 14 actions. For example, “How confident are you that you would: Express your discomfort if someone makes a joke about a woman’s body?”.

Internal consistencies (Cronbach’s alpha) of the scales for this sample were satisfying, as all were >0.75 (Table 1).

**TABLE 1:** Bivariate relations, Cronbach’s alpha, descriptive statistics for the whole sample (*N* = 3,966).

	1	2	3	4	5	6
1. System justification gender (GSJ)	–					
2. Acceptance of myth “She asked for it”	0.307**	–				
3. Acceptance of myth “He didn’t mean to”	0.228**	0.448**	–			
4. Acceptance of myth “It wasn’t really rape”	0.192**	0.480**	0.380**	–		
5. Acceptance of myth “She lied”	0.207**	0.539**	0.471**	0.550**	–	
6. Bystander intention to intervene	−0.136**	−0.322**	−0.180**	−0.224**	−0.211**	–
Cronbach’s alpha	0.77	0.80	0.78	0.86	0.90	0.83
*M*	4.39	1.84	2.22	1.42	2.06	8.13
*SD*	1.48	0.76	1.00	0.72	0.94	1.30

**p < 0.001.

### Procedure

The study is part of the USVreact (Universities Supporting Victims of Sexual Violence) project^2^ involving the University of Turin and the Politecnico di Torino, Italy, designed to develop innovative training for university staff to respond to disclosures of sexual violence on campus. The questionnaire was submitted for approval by the Bioethics Committee of the University of Turin (Approval No. 234687, 20 October 2016). We uploaded the questionnaire to the LimeSurvey platform for the online survey. After receiving approval from the rectors of the two universities, we sent an e-mail describing the research project and invited the students to complete an online questionnaire that they could open via the attached link. Before filling out the questionnaire, they were asked to read and complete the informed consent forms for data privacy. The data were collected between December 2016 and March 2017. Participation was voluntary and anonymous, and anonymity of findings was ensured. No compensation was offered for participation in the study.

### Data Analysis

The data were entered in a database and preliminarily analyzed using the IBM SPSS statistics package, Version 25, for Windows. After verifying the internal consistency of each scale and subscale with Cronbach’s alpha coefficient, we calculated the synthetic indexes (summed score of each scale). Descriptive statistics (i.e., mean and standard deviation), independent sample *T*-test, and bivariate correlations between the dimensions were performed. Effect size was then calculated by Cohen’s *d*. The standards to evaluate the strength of the effect are indicated by Cohen (1988): *d* = 0.20 is small, *d* = 0.50 is moderate, and *d* = 0.80 is large. To test the measurement model, confirmatory factor analysis (CFA) using Mplus 8 software to analyze structural equation models (maximum-likelihood method of estimation) was applied to the model that separately examined, in addition to the GSJ and bystander intention to intervene scales, the four dimensions of rape myth acceptance, and to the model that examined rape myth acceptance on a single-factor scale. The goodness of model fit of the two CFA models was compared to verify whether the four-factor structure of the rape myth acceptance scale was more suitable.

Subsequently, to verify the mediation hypothesis, the model in which the influence of GSJ on bystander intention to intervene is mediated by acceptance of the four rape myths was tested for the whole sample using the maximum-likelihood estimation method. Indexes to assess the goodness of model fit were: the chi-square value (χ^2^), the comparative fit index (CFI), the Tucker–Lewis index (TLI), the root mean square error of approximation (RMSEA), and the standardized root mean square residual (SRMR). Acceptable values are >0.90 for the CFI and the TLI (Bentler, 1995; Hoyle, 1995), <0.08 for the RMSEA (Mulaik et al., 1989), and from 0 to 1 for the SRMR. Good values are 0.95 or higher for the CFI and the TLI, 0.06 or less for the RMSEA (Hu and Bentler, 1999), and <0.05 for the SRMR (Byrne, 1998; Diamantopoulos and Siguaw, 2000; Hooper et al., 2008). The bootstrapping procedure to test the significance of the mediation test extracted 5,000 new samples from the original sample and evaluated the direct and indirect effects of the model. Mediation is significant when zero is not included in the confidence interval (Miceli, 2013). The confidence interval was set at a level of significance of.05 (CI 95%).

We examined the female and male groups separately. Correlations between dimensions for the two groups were calculated. Finally, the mediation model, evaluated for the whole sample, was tested on the female and the male sample using a multi-group full structural equation model run in Mplus 4 to analyze how the model performed in the two groups. In order to check whether the relationship between the constructs performed differently for the two groups, the constrained (forcing the pathways to be constant in the two groups) and the unconstrained model were compared via the chi-square difference test.

## Results

### Whole Sample

#### Descriptive Statistics

Descriptive statistics (Table 1) evidenced low agreement with GSJ and very low rape myth acceptance. This was true even though the myth He didn’t mean to, which minimizes the perpetrator’s responsibility, had higher agreement. Bystander intention to intervene, however, was quite high.

#### Independent Sample *T*-Test

This test highlighted significant differences between the female and the male group for all dimensions (Table 2). Specifically, the male group showed higher agreement with GSJ and with the four rape myths but lower bystander intention to intervene than the female group. Calculating Cohen’s *d*, the effect size can be considered negative moderate (from −0.52 to −0.58) for GSJ and for the acceptance of the myth “She asked for it”; positive moderate for bystander intention to intervene (0.51); and negative, between small and moderate (from −0.32 to −0.43), for acceptance of the myths “He didn’t mean to,” “It wasn’t really rape,” and “She lied.” These results confirmed hypothesis H1 that women held a higher level of bystander intention to intervene, while men showed a higher level both of GSJ and of the four dimensions of rape myth acceptance.

**TABLE 2:** Bivariate relations for female and male groups [male group (*N* = 1,142) above the diagonal, female group (*N* = 2,824) under the diagonal] and independent sample *T*-test for female and male groups.

	1	2	3	4	5	6	*M* (*SD*) Female	*M* (*SD*) Male	*T-test*	Effect size Cohen’s *d*
1. GSJ	–	0.262**	0.195**	0.185**	0.156**	−0.117**	4.18 (1.44)	4.92 (1.43)	*t*(3,964) = −14.587, *p* < 0.001	−0.52
2. Acceptance of myth “She asked for it”	0.266**	–	0.426**	0.510**	0.559**	−0.331**	1.72 (0.70)	2.16 (0.81)	*t*(1,860.411) = −16.1141, *p* < 0.001	−0.58
3. Acceptance of myth “He didn’t mean to”	0.191**	0.416**	–	0.306**	0.447**	−0.177**	2.10 (0.98)	2.53 (1.00)	*t*(3,958) = −12.598, *p* < 0.001	−0.43
4. Acceptance of myth “It wasn’t really rape”	0.156**	0.440**	0.386**	–	0.514**	−0.299**	1.36 (0.70)	1.59 (0.75)	*t*(1,995.578) = −9.129, *p* < 0.001	−0.32
5. Acceptance of myth “She lied”	0.177**	0.495**	0.452**	0.549**	–	−0.241**	1.95 (0.90)	2.35 (0.98)	*t*(1,949.907) = −11.894, *p* < 0.001	−0.43
6. Bystander intention to intervene	−0.073**	−0.245**	−0.122**	−0.142**	−0.137**	–	8.32 (1.14)	7.65 (1.48)	*t*(1,722.097) = 13.870, *p* < 0.001	0.51

**p < 0.001.

#### Bivariate Correlations

The dimensions were all significantly related (Table 1). Specifically, acceptance of each of the four rape myths strictly positively related to each other and directly correlated with GSJ. In particular, the myth “She asked for it” had a strong relation with GSJ; the myths “He didn’t mean to” and “She lied” were quite strongly related to GSJ; while the relation of the myth “It wasn’t really rape” with GSJ was less intense. Rape myth acceptance is coherent with the belief that relationships between men and women are fair. In contrast, these dimensions are inversely related with bystander intention to intervene. Specifically, the myth “She asked for it” has a strong, inverse relation with bystander intention to intervene; the myths “It wasn’t really rape” and “She lied” had a quite intense inverse relation with bystander intervention attitude to intervene; and the myth “He didn’t mean to” as GSJ had a less strong inverse relation with bystander intention to intervene. In other words, if a respondent endorsed GSJ and rape myths, he/she less likely intends to intervene as a bystander. Hypothesis H2, that the four dimensions of rape myth acceptance are positively related to each other and to GSJ, while bystander intention to intervene has a negative relation both with the acceptance of each of the four rape myths and with GSJ, was thus confirmed.

#### Measurement Models

We compared the two measurement models where, in addition to the single-factor scale of GSJ and of bystander intention to intervene, we examined the scale of rape myth acceptance on a four-factor and single-factor basis. The model that analyzed the four dimensions of rape myth acceptance separately showed the best fit indexes [one-factor: χ^2^(811) 14,253.161, *p* < 0.001, CFI = 0.809, TLI = 0.797, RMSEA = 0.065 (0.064, 0.066), and SRMR 0.060; four-factor: χ^2^(799) 6,022.275, *p* < 0.001, CFI = 0.926, TLI = 0.920, RMSEA = 0.041 (0.040, 0.042), and SRMR 0.043].

#### Structural Equation Mediation Models

**FIGURE 1:**
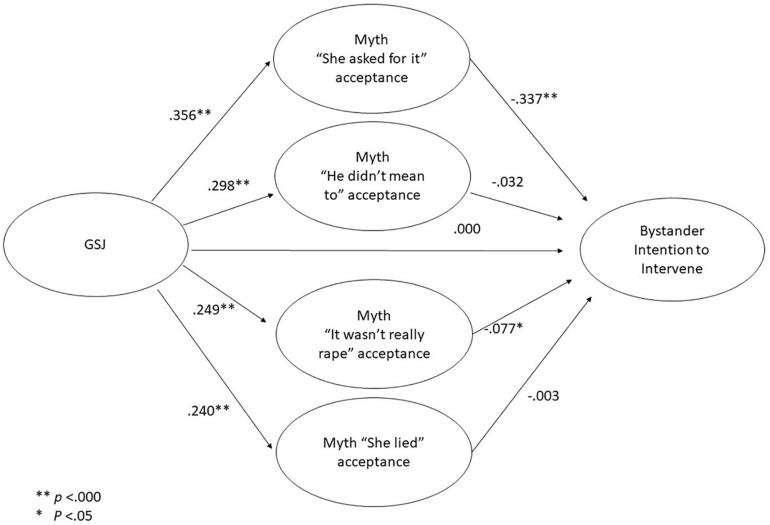
Structural equation models of mediation between gender-specific system justification (GSJ) and bystander intention to intervene by rape myth acceptance for the whole sample.

In order to confirm the hypothesis that rape myth acceptance mediates the relationship between GSJ and bystander intention to intervene, the model in which GSJ influence on bystander intention to intervene is mediated by acceptance of the four rape myths was tested using Mplus 8 software. The model (Figure 1) showed that acceptance of the four rape myths had a significant relationship with GSJ: acceptance of the rape myth “She asked for it” had a significant relation with bystander intention to intervene (*p* < 0.001), as did acceptance of the myth “It wasn’t really rape” (*p* = 0.044), whereas acceptance of the rape myths “He didn’t mean to” and “She lied” were not related to bystander intention to intervene. GSJ had no direct link with bystander intention to intervene.

The model showed satisfactory indexes of fit: CFI = 0.938, TLI = 0.933, RMSEA = 0.037 (0.036, 0.038), and SRMR 0.042. Only the chi-square value of the model was quite high [χ^2^(797) 5,159.52, *p* < 0.001], but the significance of χ^2^ depends on the sample size, and our sample was quite large (*N* = 3,866).

The bootstrapping procedure to evaluate the direct and indirect effects of rape myth acceptance (Table 3) highlighted that only the mediated effect for acceptance of the rape myth “She asked for it” was statistically significant for the whole sample, as zero was not comprised in the CI (95%), while the mediated effect for acceptance of the rape myth “It wasn’t really rape” was not significant (significance level of 0.05). Other mediated effects and the direct effect of GSJ on bystander intention to intervene were not statistically significant, as they were comprised in the CI (95%). We can state that the hypothesis (H3) of mediation was partially confirmed, as only acceptance of the rape myth “She asked for it” mediated between GSJ and bystander intention to intervene. Acceptance of the two rape myths “He didn’t mean to” and “She lied” did not mediate the relationship between GSJ and bystander intention to intervene, and acceptance of the myth “It wasn’t really rape” had a significant link with bystander intention to intervene but no significant indirect effect.

**TABLE 3:** Evaluation of indirect and direct effects using bootstrapping procedure (5,000 extractions) in model for whole sample, for female group, and for male group.

		Est.	SE	*p*	CI 95%
**Whole sample**	*Indirect effect*				
	GSJ→“She asked for it”→Byst. Int. to Int.	−0.120	0.013	<0.001	−0.145, −0.095
	GSJ→“He didn’t mean to”→ Byst. Int. to Int.	−0.010	0.009	0.261	−0.027, 0.007
	GSJ→“It wasn’t really rape”→ Byst. Int. to Int.	−0.019	0.010	0.049	−0.038, <0.001
	GSJ→“She lied”→ Byst. Int. to Int.	−0.001	0.007	0.928	−0.015, 0.014
	*Direct effect*				
	GSJ→Byst. Int. to Int.	<0.001	0.023	0.999	−0.045, 0.045
**Female group**	*Indirect effect*				
	GSJ→“She asked for it”→Byst. Int. to Int.	−0.096	0.013	0.000	−0.121, −0.071
	GSJ→“He didn’t mean to”→Byst. Int. to Int.	−0.006	0.009	0.505	−0.023, 0.011
	GSJ→“It wasn’t really rape”→Byst. Int. to Int.	−0.006	0.009	0.484	−0.024, 0.011
	GSJ→“She lied”→Byst. Int. to Int.	0.003	0.007	0.657	−0.011, 0.017
	Direct effect				
	GSJ→ Byst. Int. to Int.	0.026	0.026	0.318	−0.025, 0.076
**Male group**	*Indirect effect*				
	GSJ→“She asked for it”→Byst. Int. to Int.	−0.085	0.025	0.001	−0.135, −0.035
	GSJ→“He didn’t mean to”→Byst. Int. to Int.	−0.006	0.015	0.696	−0.036, 0.024
	GSJ→“It wasn’t really rape”→Byst. Int. to Int.	−0.057	0.025	0.020	−0.105, −0.009
	GSJ→“She lied”→Byst. Int. to Int.	−0.001	0.011	0.934	−0.023, 0.022
	*Direct effect*				
	GSJ→Byst. Int. to Int.	0.016	0.044	0.708	−0.069, 0.102

### Female and Male Groups Examined Separately

#### Bivariate Correlations

Examination of the two groups separately revealed that the relationships between the dimensions were quite similar to the correlation patterns observed for the whole sample, though we noted generally stronger correlations among the dimensions for the male sample (Table 2).

#### Multi-Group Structural Equation Mediation Model

The independent sample *T*-test showed marked differences between men and women for all considered variables. Using Mplus 8 software, we analyzed a multi-group structural equation model comparing the female and the male group to determine whether relationships among the constructs worked in different ways. Indexes of fit of the multi-group mediation model were satisfactory (Table 4). Only the chi-square value of the model was quite high, as the sample was very large. The chi-square difference test comparing the constrained model M_0_, in which the mediation paths for the female and the male group were forced to be the same as in the unconstrained model M_1_, showed a significant difference (Table 4): the mediation model did not perform in the same way for males and females.

**TABLE 4:** Comparison between constrained and unconstrained model, for female and for male groups, in multi-group mediation model.

	χ^2^	*df*	*p*	CFI	TLI	RSMSEA	SRMR	Δχ^2^	*P*
M_0_	7,104.033	1,675	<0.001	0.920	0.918	0.040 (0.039, 0.041)	0.050		
M_1_	7,070.260	1,666	<0.001	0.921	0.918	0.040 (0.039, 0.041)	0.048	33.773	<0.01

*M_0_, constrained model where mediation paths for female and male groups were forced to be the same; M_1_, unconstrained model; CFI, comparative fit index; TLI, Tucker–Lewis index; RMSEA, root mean square error of approximation; SRMR, standardized root mean square residual.*

**FIGURE 2:**
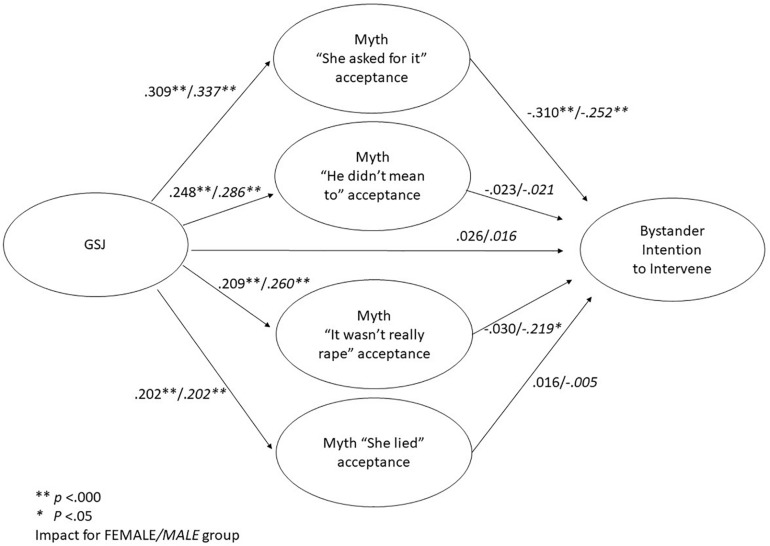
Multi-group structural equation models of mediation by rape myth acceptance between GSJ and bystander intention to intervene for the female and the male group.

Figure 2 presents the mediation models for the female and the male group. In both groups, acceptance of the four rape myths was significantly influenced by GSJ, whereas bystander intention to intervene had no direct link with GSJ and acceptance of the myths “He didn’t mean to” and “She lied.” In the female group, bystander intention to intervene was significantly negatively related only to acceptance of the myth “She asked for it,” while in the male group, acceptance of the myths “She asked for it” and “It wasn’t really rape” had a significant link with bystander intention to intervene. Mediation between GSJ and bystander intention to intervene by rape myth acceptance was confirmed in the two groups, but acceptance of the myth “It wasn’t really rape” mediated this relationship only for the male group.

The bootstrapping procedure to evaluate direct and indirect effects (Table 3) separately for the female and the male group confirmed that the mediated effect for acceptance of the rape myth “She asked for it” was statistically significant for both groups: zero was not comprised in the CI (95%). The mediated effect for acceptance of the rape myth “It wasn’t really rape,” however, was significant only for the male group. Other mediated effects and the direct effect of GSJ on bystander intention to intervene were not significant in either group, as zero was comprised in the CI.

## Discussion

This study had three main aims: (1) to analyze the relationships between GSJ, the four dimensions of rape myth acceptance, and bystander intention to intervene in an Italian sample of 3,966 university students; (2) to explore whether the four dimensions of rape myth acceptance play different roles in the relationships; and (3) to explore how the relationships differ for the male and the female group.

One of this study’s central findings indicates that there are gender differences in system justification gender and in myth acceptance. Men held significantly higher levels than women of GSJ and acceptance of the four rape myths, whereas the females were more disposed than males to bystander intervention against sexual assault, fully confirming our initial hypotheses. These findings are in line with previous studies in other cultural contexts (e.g., Chapleau and Oswald, 2014; Navarro and Tewksbury, 2017) and suggest that even though women belong to the same cultural context as men, they more easily identify with the victim or, at least, with the penalized group in the game of gender relationships. As most sexual assaults are toward females (World Health Organization [WHO] and Human Reproduction Programme [HRP], 2019), women fear themselves becoming victims of rape. Identification with the victim decreases the probability of developing victim blaming (Hayes et al., 2013). In line with the Shaver’s (1970) defensive attribution theory, indeed, as women feel similarity with the victims, they tend to engage in victim blaming less than men do (Langhinrichsen-Rohling et al., 2004; Grubb and Harrower, 2008).

Gender-specific system justification and acceptance of the four rape myths did not show higher medium values, with no alarming sexual assault supportive attitudes among students toward sexual violence and quality of gender relationships. The hypothesis that the four dimensions of rape myth acceptance are positively related to each other, that GSJ is positively related with all four dimensions of rape myth acceptance and negatively related to bystander intention to intervene, and that all four dimensions of rape myth acceptance are negatively related to bystander intention to intervene, notwithstanding, was also verified by bivariate correlations. GSJ and acceptance of the four rape myths, indeed, had high direct relations to each other and inverse strong relations with bystander intention to intervene, consistent with several previous studies (e.g., McMahon, 2010; Chapleau and Oswald, 2014). System justification and rape myth acceptance are attitudes that are expressions of a patriarchal social system that safeguards the prestige of the privileged group (males) (Jost and Banaji, 1994; Jost and Kay, 2005; Bolton et al., 2018) and of an ideology that justifies women’s subordination (Stoll et al., 2017). Our study supports the idea that the belief in a right and fair social system governing gender relationships (system justification gender) is related to justification and/or underestimation of sexual violence (Ståhl et al., 2010; Chapleau and Oswald, 2013, 2014), and both can affect the intention to intervene.

In addition, the hypothesis that the influence of GSJ on bystander intention to intervene is fully mediated by the four dimensions of rape myth acceptance was partially confirmed: the structural equation model showed a mediation role of acceptance of the rape myth “She asked for it” between GSJ and bystander intention to intervene. Specifically, our results evidenced that GSJ had a significant relation with acceptance of the four rape myths, while bystander intention to intervene had a significant negative relation only with acceptance of the myth “She asked for it” and no relation with acceptance of the three other myths “He didn’t mean to,” “It wasn’t really rape,” and “She lied.” GSJ, then, had no direct link with bystander intention to intervene. Our findings show a significant impact of acceptance of the myth “She asked for it” in reducing bystander intention to intervene, confirming the tendency to attribute the absence of interventions to the victim’s blame. This type of blame assignment cannot be underestimated. The belief that the victim somehow “provoked” the sexual assault is a myth entrenched in the current social context, even among younger generations and in both men and women. As Barnes et al. (1979) demonstrated over 40 years ago, one of the factors that can foster or inhibit helpful behavior is whether one considers the victim responsible for the situation that he or she is in. In matters of social aggression, gender stereotypes “legitimize” blaming the victim for being assaulted. In other words, respondents tended to have low bystander intention to intervene and to blame the victim for being sexually assaulted when they sought to justify the current gender relations system. Also, they may tend not to intervene as a bystander when they do not recognize a situation as involving sexual assault.

Our data underline the importance of the role of blaming (“She asked for it”) and of the perception of what is/isn’t violence (“It wasn’t really rape”) in the intention to intervene, whereas “He didn’t mean to” and “She lied” do not have a mediator role. These two myths may refer to a dispositional attribution about the two subjects involved: the woman, a liar, and a man, who shares a common sense of what can be justified. We assume that they are a form of *ex post* justification for sexual assault, less important for the bystander’s evaluation of the situation and his/her decision to intervene. Further analysis will be useful to better explain the roles and the effects of acceptance of rape myths. Moreover, since GSJ had a significant relationship with acceptance of all four rape myths, we can state that the different dimensions of rape myth acceptance play different roles in mediating the relationship. Consistent with the work of Taschler and West (2017) and McMahon and Farmer (2011), this result substantiates the importance of analyzing acceptance of the different rape myths separately rather than as a whole construct. Indeed, the measurement model of four-factorial rape myth acceptance showed better indices of fit than the single-factor model.

Moreover, in answer to our explorative question about how the mediation model performed in the female and the male groups, our findings showed that gender differences need to be included in the analysis of the relationship between GSJ, rape myth acceptance, and bystander intention to intervene. The correlations between the different dimensions were generally stronger for the men, and the results of the mediation model differed between the two groups. Only acceptance of the myth “She asked for it” played a significant mediation role between GSJ and bystander intention to intervene for the women, whereas for the men, there was a significant mediated effect for acceptance of the rape myths “She asked for it” and “It wasn’t really rape.” In other words, both groups tended to have low bystander intention to intervene and to blame the victim for being sexually assaulted when the groups sought to justify the current gender relations system. Specifically, men appear less disposed to intervene when they underestimate, or do not recognize, a behavior as “violence.”

Finally, though women held a lower level of rape myth acceptance than men did, the attitude of blaming the victim (the myth “She asked for it”) has different functions for males and females. For men, it could mean defending their in-group status, justifying the perpetrator, or perceiving the act as not so harmful or serious in its consequences. Differently for women, it could serve a defensive function, because if the responsibility is assigned to the victim and not to the socio-normative context, women can believe it is possible to avoid falling victim to the same situation by avoiding the same type of behavior.

### Limitations and Future Directions

The present study has several limitations. Although the sample was numerically quite large, all respondents came from the same geographic area. As the socio-cultural context may affect such dimensions as GSJ and rape myth acceptance, future studies should involve respondents from several different geographical areas. The survey respondents were all university students. Although previous studies (e.g., McMahon and Farmer, 2011; Chapleau and Oswald, 2014; Bendixen and Kennair, 2017) have used similar samples, it is important for future research to involve participants of different age groups and education levels in order to obtain generalizable study results. Another potential weakness is that, because the data were cross-sectional, we were unable to assess causality in the relationships between variables. To this end, we are planning a second wave to acquire longitudinal data. Moreover, future research should analyze the relationships between GSJ, rape myth acceptance, bystander intention to intervene, and other factors such as the relationships between these constructs and the commodity model of sex. This may be particularly important, as Johnson (2015) observed that bystander intention to intervene is predicted by rape myth acceptance and that rape myth acceptance is, in turn, predicted by adherence to the commodity model of sex. The commodity model of sex states that a culturally widespread conception of sexual relations, in a patriarchal matrix, represents sex as a property: something that can “be given, bought, sold, or stolen […] women have it, and men try to get it” (Millar, 2008, p. 30; in Johnson, 2015, p. 11). Other variables that could enrich the analysis of these phenomena are the belief in a just world and sexism. Belief in a just world (Lerner, 1980; Hammond et al., 2011), indeed, could be strongly threatened by considering gender violence unjust: to reduce cognitive discomfort (dissonance), witnesses to violence and even victims often tend to minimize its harm or justify it. Diener (2016) used separate hierarchical regression models to show that belief in a just world and sexism predict rape myth acceptance and that belief in a just world and sexism influence bystander intention to intervene. Finally, exploring gender-related differences in attitudes toward sexual violence and intervention against it is important for their theoretical and practical implications.

Adolfsson and Strömwall (2017) suggested that other variables come into play in blame attribution and that gender in itself cannot fully explain these differences. They state, for example, that gender equality in a given country could also affect gender differences in assigning blame to the victim. Chapleau and Oswald (2014) reported that “the association between national levels of rape myth acceptance and gender inequality to our knowledge has yet to be examined” (p. 205), and they emphasized that “rape myth acceptance justifies a system of gender inequality” (p. 205). Furthermore, an intersectional perspective (Crenshaw, 1989; Bose, 2012) could yield further information for a deeper analysis.

### Research and Policy Implications

Although our data do not show high levels of rape myth acceptance, we cannot ignore the persistence of false beliefs related to gender violence, even in a sample of young university students. It is therefore necessary to develop education and training courses and to raise awareness in the community of the urgent need for intervention. It has been shown, however, that prevention programs “presenting factual information about sexual assault that contradicts rape myths does not appear sufficient for lasting change” (Joseph et al., 2013, p. 500). Before efforts can be undertaken to increase bystander intentions to intervene, it is necessary to understand the “functions served by rape myths (e.g., allow women to maintain their sense of safety by distancing themselves from the female victim stereotype, provide men with guidelines for behavior in sexually ambiguous situations)” (ibidem). The results of the present work can be used to inform training programs that focus on changing attitudes that seem to inhibit bystander intervention, by trying not only to reduce victim blaming and to help people to recognize forms of violence, but also to eradicate GSJ.

## Data Availability Statement

The datasets generated for this study are available on request to the corresponding author.

## Ethics Statement

All procedures performed in studies involving human participants were in accordance with the tenets of the 1964 Helsinki Declaration and its later amendments or comparable Ethical Statements and with the Ethical Standards of the Bioethics Committee of University of Turin (Approval No. 234687, 20 October 2016). The participants provided their written informed consent to participate in this study.

## Author Contributions

MM and ND developed the design of the manuscript and wrote theoretical sections “Introduction” and “Discussion.” MM developed moreover the sections “Materials and Methods” and “Results.”

## Conflict of Interest

The authors declare that the research was conducted in the absence of any commercial or financial relationships that could be construed as a potential conflict of interest.

## Footnotes

1 Martini, M., Tartaglia, S., and De Piccoli, N. (under review). Assessing rape myth acceptance: a contribution to italian validation of the measure for assessing subtle rape myth (SRMA-IT).

2 “USVreact, Universities Supporting Victims of Sexual Violence: Training for Sustainable Services” (project code: JUST/2014/RDAP/AG/VICT/7401; duration: March 2016–2018; website: http://usvreact.eu/). Funded with support from the European Commission’s Directorate-General (DG) Justice, Rights, Equality and Citizenship Programme (DAPHNE strand). The European Commission cannot be held responsible for any use that may be made of the information contained therein.
